# Improving Frontline Healthcare Workers' Knowledge of Japanese Encephalitis and Other Vector-Borne Diseases Through a One-Day Interactive Training Programme: A Quasi-experimental Study

**DOI:** 10.7759/cureus.113491

**Published:** 2026-07-28

**Authors:** Bijit Biswas, Gurusanganabasava Hawaldar, G. Jahnavi

**Affiliations:** 1 Community and Family Medicine, All India Institute of Medical Sciences, Deoghar, IND

**Keywords:** community health workers, encephalitis, frontline healthcare workers, india, inservice training, japanese, japanese encephalitis, knowledge, vector-borne diseases

## Abstract

Background: Frontline healthcare workers (FHWs) are central to vector-borne disease (VBD) prevention and control, but evidence on the effectiveness of routine training programmes remains limited. This study evaluated the effectiveness of a one-day interactive training programme among FHWs in eastern India.

Methods: A quasi-experimental single-group pre-test-post-test study was conducted among FHWs in Deoghar, Jharkhand, India. Auxiliary nurse midwives (ANMs) and multi-purpose workers (MPWs) (n = 39) received training on Japanese encephalitis (JE), while community health officers (CHOs) (n = 38) received training on major VBDs. Knowledge was assessed immediately before and after training using validated questionnaires.

Results: Mean JE knowledge scores increased from 7.8 ± 3.8 to 17.1 ± 2.8 (mean difference: 9.3; Cohen's d = 1.9; p < 0.001) among ANMs and MPWs, while mean VBD knowledge scores increased from 8.5 ± 4.0 to 15.9 ± 2.6 (mean difference: 7.0; Cohen's d = 1.7; p < 0.001) among CHOs. Correct responses increased significantly for 18 of 20 JE-related knowledge items and 17 of 18 VBD-related knowledge items. Educational level independently predicted greater JE knowledge gain (B = 1.03, 95% CI: 0.34-1.72; p = 0.005), whereas no participant characteristic was associated with knowledge gain among CHOs.

Conclusions: A structured one-day interactive training programme substantially improved knowledge of JE and other major VBDs among FHWs. These findings support regular guideline-based refresher training to strengthen frontline capacity for VBD prevention and control.

## Introduction

Vector-borne diseases (VBDs) remain a major public health challenge, accounting for more than 17% of all infectious diseases and causing over 700,000 deaths annually worldwide [1]. India bears a substantial burden of malaria, dengue, chikungunya, Japanese encephalitis (JE), lymphatic filariasis (LF), and *kala-azar* (KA; visceral leishmaniasis). Although sustained control programmes have substantially reduced the burden of malaria, LF, and KA, dengue and chikungunya continue to cause frequent outbreaks, while JE remains endemic in several states, with more than 2,500 cases and over 500 deaths reported annually [2-6]. These diseases continue to pose considerable public health challenges despite ongoing control efforts [7,8].

The National Center for Vector Borne Diseases Control (NCVBDC) is responsible for implementing prevention, control, and elimination strategies for these diseases through a network of frontline healthcare workers (FHWs), including auxiliary nurse midwives (ANMs), multi-purpose workers (MPWs), and community health officers (CHOs). These healthcare workers play a pivotal role in disease surveillance, early case detection, referral, implementation of vector control measures, health education, and community mobilization. Their effectiveness depends on adequate knowledge of evolving epidemiology, national guidelines, and programme strategies, making regular capacity-building and refresher training essential [9-11].

Although training programmes are routinely conducted under the NCVBDC, published evidence evaluating their effectiveness in improving knowledge among FHWs remains limited, particularly in resource-constrained settings. Therefore, this study evaluated the effectiveness of one-day interactive training programmes on JE and other major VBDs in improving the knowledge of FHWs in Deoghar district, Jharkhand, India.

## Materials and methods

Study design and setting

A quasi-experimental single-group pre-test-post-test study was conducted among FHWs in Deoghar district, Jharkhand, India, during September and October 2025. Two routine training programmes were organized at the District Hospital, Deoghar, under the Civil Surgeon-cum-Chief Medical Officer's Office following a request from the NCVBDC, Jharkhand.

Study participants and sampling

Convenience sampling was used. The study included all participants selected by the NCVBDC for the routine training programme who attended the complete training and completed both the pre- and post-training assessments. The first training comprised ANMs and MPWs (n = 39) receiving training on JE, while the second comprised CHOs (n = 38) receiving training on major VBDs, including malaria, dengue, LF, KA, and JE.

Sample size

The minimum sample size was estimated using Statulator [12] assuming an effect size of 0.5, 80% power, and a two-sided significance level (α) of 5%, yielding a required sample size of 34 participants. Both training groups exceeded the minimum required sample size.

Educational intervention

Two separate one-day interactive training programmes were delivered by faculty from the Department of Community and Family Medicine (CFM), All India Institute of Medical Sciences (AIIMS), Deoghar, using standardized bilingual (English-Hindi) PowerPoint presentations developed according to the latest NCVBDC, Government of India operational guidelines and training materials [9-11,13-16]. The JE training for ANMs and MPWs covered the epidemiology, transmission, clinical features, surveillance, diagnosis, specimen collection and transport, case management, referral, vaccination, and prevention of JE and acute encephalitis syndrome (AES). The VBD training for CHOs focused on the epidemiology, surveillance, diagnosis, treatment, vector control, programme implementation, and elimination strategies for malaria, dengue, LF, KA, and JE. Each training programme consisted of a 15-minute pre-training assessment, a 2.5-hour interactive session comprising lectures, audiovisual presentations, facilitated discussions, case-based learning, participant feedback, and question-and-answer sessions, followed by a 15-minute post-training assessment.

Data collection

Knowledge was assessed using structured self-administered questionnaires administered through Google Forms (Google, Mountain View, CA) immediately before and after each training programme. The questionnaires were developed by the investigators and based on the latest NCVBDC operational guidelines and Government of India training modules. The JE and VBD questionnaires comprised 20 and 18 multiple-choice items, respectively. Internal consistency was acceptable, with Cronbach's alpha values of 0.757 (pre-training) and 0.740 (post-training) for the JE questionnaire, and 0.777 (pre-training) and 0.782 (post-training) for the VBD questionnaire. Each correct response was awarded one mark, while incorrect or unanswered responses received zero marks, yielding total attainable knowledge scores ranging from 0 to 20 for the JE questionnaire and 0 to 18 for the VBD questionnaire. Sociodemographic and professional characteristics, including age, gender, educational level, years of service, place of posting, and occupation, were also collected.

Ethics approval and consent to participate

The study (IEC Ref. No. 2025-531-IND-05) was approved by the Institutional Ethics Committee, AIIMS, Deoghar, Jharkhand, India. Administrative permission to conduct the training programmes was obtained from the Civil Surgeon-cum-Chief Medical Officer, Deoghar, Jharkhand. The study was conducted in accordance with the ethical principles of the Declaration of Helsinki and its subsequent amendments. Written informed consent was obtained from all participants before enrolment. Participation was voluntary, and participant confidentiality, privacy, and anonymity were maintained throughout data collection, analysis, interpretation, and reporting.

Statistical analysis

Data were analysed using Jamovi (version 2.6.44). Continuous variables were presented as mean ± standard deviation (SD) or median with interquartile range (IQR), as appropriate, whereas categorical variables were presented as frequencies, percentages, and 95% confidence intervals (CIs). Item-wise changes in paired responses were analysed using McNemar's chi-square test. Changes in overall knowledge scores before and after training were evaluated using the paired t-test, and effect sizes were quantified using Cohen's d with 95% CIs. Associations between participant characteristics and change in knowledge score (post-training score minus pre-training score) were assessed using Spearman's rank correlation. Multiple linear regression analysis was performed among ANMs and MPWs to identify independent predictors of knowledge gain. Regression assumptions were assessed using variance inflation factors (VIFs) to evaluate multicollinearity and Cook's distance to identify influential observations. All statistical tests were two-sided, and a p-value <0.05 was considered statistically significant.

## Results

Among ANMs and MPWs receiving the JE training, the median age was 39 years (IQR: 34-46; range: 24-59 years). Most participants were female (64.1%). The median educational level was 12 years (IQR: 12-15; range: 10-19 years), and the median duration of work experience was 12 years (IQR: 8-16; range: 1-37 years). Participants were predominantly posted at health sub centres (HSCs) (43.6%), followed by community health centres (CHCs) (41.0%) and primary health centres (PHCs) (15.4%). Most participants were ANMs (61.5%), while 38.5% were MPWs.

Among CHOs receiving the VBD training, the median age was 27 years (IQR: 26-31; range: 24-39 years). Most participants were female (71.1%). The majority held a Bachelor of Science (B.Sc.) in nursing degree (60.5%), while the remainder had a General Nursing and Midwifery (GNM) qualification (39.5%). The median duration of work experience was one year (IQR: 1-5; range: 1-6 years). All participants were CHOs posted at HSCs (Table 1).

**Table 1:** Background characteristics of the study participants. Abbreviations: ANM, auxiliary nurse midwife; CHC, community health centre; CHO, community health officer; CI, confidence interval; HSC, health sub-centre; MPW, multi-purpose worker; PHC, primary health centre; BSc, Bachelor of Science; GNM, General Nursing and Midwifery.

Variable	n (%)	95% CI
Training 1. Japanese encephalitis (ANMs and MPWs; n = 39)		
Age in completed years		
<30	5 (12.8)	5.6, 26.7
30-39	14 (35.9)	22.7, 51.6
40-49	14 (35.9)	22.7, 51.6
50-59	6 (15.4)	7.2, 29.7
Gender		
Male	14 (35.9)	22.7, 51.6
Female	25 (64.1)	48.4, 77.3
Educational level		
Secondary	6 (15.4)	7.2, 29.7
Higher secondary	16 (41.0)	27.1, 56.6
Graduate and above	17 (43.6)	29.3, 59.0
Occupation		
ANM	24 (61.5)	45.9, 75.1
MPW	15 (38.5)	24.9, 54.1
Place of posting		
HSC	17 (43.6)	29.3, 59.0
PHC	6 (15.4)	7.2, 29.7
CHC	16 (41.0)	27.1, 56.6
Years of experience		
<10	11 (28.2)	16.5, 43.8
10-14	15 (38.5)	24.9, 54.1
15-19	8 (20.5)	10.7, 35.5
≥20	5 (12.8)	5.6, 26.7
Training 2. Vector-borne disease (CHOs; n = 38)		
Age in completed years		
≤25	9 (23.7)	12.9, 39.2
26-30	19 (50.0)	34.8, 65.1
31-35	7 (18.4)	9.2, 33.4
≥36	3 (7.9)	2.7, 20.8
Gender		
Male	11 (28.9)	17.0, 44.7
Female	27 (71.1)	55.2, 83.0
Education		
BSc	23 (60.5)	44.7, 74.4
GNM	15 (39.5)	25.6, 55.3
Years of experience		
1	25 (65.8)	49.8, 78.8
2	2 (5.3)	1.5, 17.3
≥3	11 (29.9)	17.0, 44.8

The educational intervention significantly improved knowledge across almost all assessed items in both training groups. Correct responses increased significantly for 18 of the 20 JE-related knowledge items. K2 (JE virus family) did not improve significantly (p = 0.064), while the p-value for K6 (JE virus incubation period) could not be calculated because all paired responses were concordant after training. Among CHOs, correct responses increased significantly for 17 of the 18 VBD-related knowledge items, while the p-value for K9 (treatment regimen for adult *Plasmodium vivax* malaria) could not be calculated because all paired responses were concordant after training (Table 2).

**Table 2:** Item-wise comparison of correct responses before and after the one-day interactive training on vector-borne disease knowledge among frontline healthcare workers. Abbreviations: ABER, annual blood examination rate; ACT, artemisinin combination therapy; AES, acute encephalitis syndrome; ANM, auxiliary nurse midwife; API, annual parasite incidence; CBC, complete blood count; CHO, community health officer; CSF, cerebrospinal fluid; DEC, diethylcarbamazine; JE, Japanese encephalitis; LF, lymphatic filariasis; MCPG, methylenecyclopropylglycine; MF, microfilaria; MPW, multi-purpose worker; PCR, polymerase chain reaction; PHC, primary health centre; ssRNA, single-stranded RNA. * P-values were calculated using McNemar's chi-square test. † P-value could not be calculated because all paired responses were concordant after training.

Item number	Knowledge item	Correct answer	Pre- training, n (%)	Post-training, n (%)	P-value^*^
Training 1. Japanese encephalitis (ANMs and MPWs; n = 39)					
K1	Main vector of JE:	Culex	25 (64.1)	38 (97.4)	0.001
K2	Family of JE virus:	Flaviviridae	20 (51.3)	29 (74.4)	0.064
K3	Genetic material of JE virus:	ssRNA	7 (17.9)	32 (82.1)	<0.001
K4	Amplifier host in JE transmission:	Pig	29 (74.4)	37 (94.9)	0.021
K5	Role of humans in JE transmission:	Dead-end host	10 (25.6)	38 (97.4)	<0.001
K6	Incubation period of JE virus:	4-14 days	24 (61.5)	39 (100.0)	^†^
K7	JE infection progressing to encephalitis:	1 in 250	12 (30.8)	38 (97.4)	<0.001
K8	AES outbreaks in litchi-producing regions linked to:	MCPG toxin	12 (30.8)	36 (92.3)	<0.001
K9	Basis for identifying suspected AES case:	Acute fever, altered sensorium, new seizures	18 (46.2)	37 (94.9)	<0.001
K10	Prodromal symptoms of JE:	Fever, headache, malaise	22 (56.4)	36 (92.3)	0.001
K11	AES danger signs include:	Unconsciousness, seizures, altered sensorium	18 (46.2)	35 (89.7)	<0.001
K12	Assertion: A small proportion of JE progress to encephalitis. Reason: Humans have low and short-lasting viremia.	Both assertion and reason are true, and reason correctly explains the assertion	5 (12.8)	21 (53.8)	<0.001
K13	Late sequelae in JE patients:	Neurological disability, behavioural problems, cognitive impairment	10 (25.6)	30 (76.9)	<0.001
K14	Intranasal Midazolam dosing during convulsions in an AES patient:	1 spray in each nostril	11 (28.2)	33 (84.6)	<0.001
K15	Adequate serum sample volume for JE testing:	1-2 ml	7 (17.9)	30 (76.9)	<0.001
K16	Adequate CSF sample volume for JE testing:	0.5-1 ml	20 (51.3)	32 (82.1)	0.012
K17	Mandatory PHC investigations for AES:	Blood glucose, CBC, malaria test	16 (41.0)	29 (74.4)	0.004
K18	Lab confirmation of JE can be done by:	Serum/CSF IgM antibody, paired sera IgG four-fold rise, PCR nucleic acid detection	13 (33.3)	28 (71.8)	0.003
K19	Position during referral to reduce aspiration risk:	Lateral position	9 (23.1)	36 (92.3)	<0.001
K20	Correct pair of Do’s and Don’ts for AES prevention:	Do: Keep pigs away from homes and cover pigpens. Don’t: Use pond/lake water for drinking	15 (38.5)	31 (79.5)	0.001
Training 2. Vector-borne disease (CHOs; n = 38)					
K1	Full form of ACT:	Artemisinin Combination Therapy	19 (50.0)	32 (84.2)	0.001
K2	Treatment regimen for mixed malaria infection:	ACT - 3 days + Primaquine - 14 days	20 (52.6)	34 (89.5)	0.004
K3	API is an indicator of:	Malaria	18 (47.4)	32 (84.2)	0.001
K4	Full form of ABER:	Annual Blood Examination Rate	26 (68.4)	35 (92.1)	0.012
K5	Symptoms can be seen in a JE suspect:	Fever, headache, convulsions, unconsciousness	22 (57.9)	35 (92.1)	0.002
K6	Drugs recommended for MF-positive case (new guidelines):	DEC + Albendazole	21 (55.3)	35 (92.1)	0.001
K7	Follow-up period for a microfilaria-positive patient:	12 months	4 (10.5)	27 (71.1)	<0.001
K8	Stage after which deep skin folds appear in LF:	Stage 3	13 (34.2)	30 (78.9)	<0.001
K9	Drug, dose & duration for treatment of adult P. vivax patient:	Chloroquine - 3 days + Primaquine - 14 days	19 (50.0)	38 (100.0)	^†^
K10	Vector and parasite causing cerebral malaria:	Anopheles - Plasmodium falciparum	24 (63.2)	36 (94.7)	0.004
K11	Definition of a positive house in dengue survey:	House where larvae are found	19 (50.0)	35 (92.1)	<0.001
K12	House Index for 1140 positive houses out of 6820:	16%	24 (63.2)	37 (97.4)	<0.001
K13	Incentive given for testing & complete treatment of a malaria patient:	₹200	16 (42.1)	33 (86.8)	<0.001
K14	Full scientific name of JE vector:	Culex tritaeniorhynchus	18 (47.4)	27 (71.1)	0.035
K15	Dengue vectors breed in:	Clean stagnant water	21 (55.3)	36 (94.7)	<0.001
K16	Criteria for suspecting kala-azar:	Fever > 2 weeks with weight loss & spleen/liver enlargement	20 (52.6)	34 (89.5)	0.001
K17	Confirmation test for kala-azar:	rk39 Rapid Diagnostic Test	19 (50.0)	33 (86.8)	0.001
K18	Recommended treatment for confirmed kala-azar:	Liposomal Amphotericin B 10 mg/kg IV, single dose	16 (42.1)	36 (94.7)	<0.001

**Figure 1:**
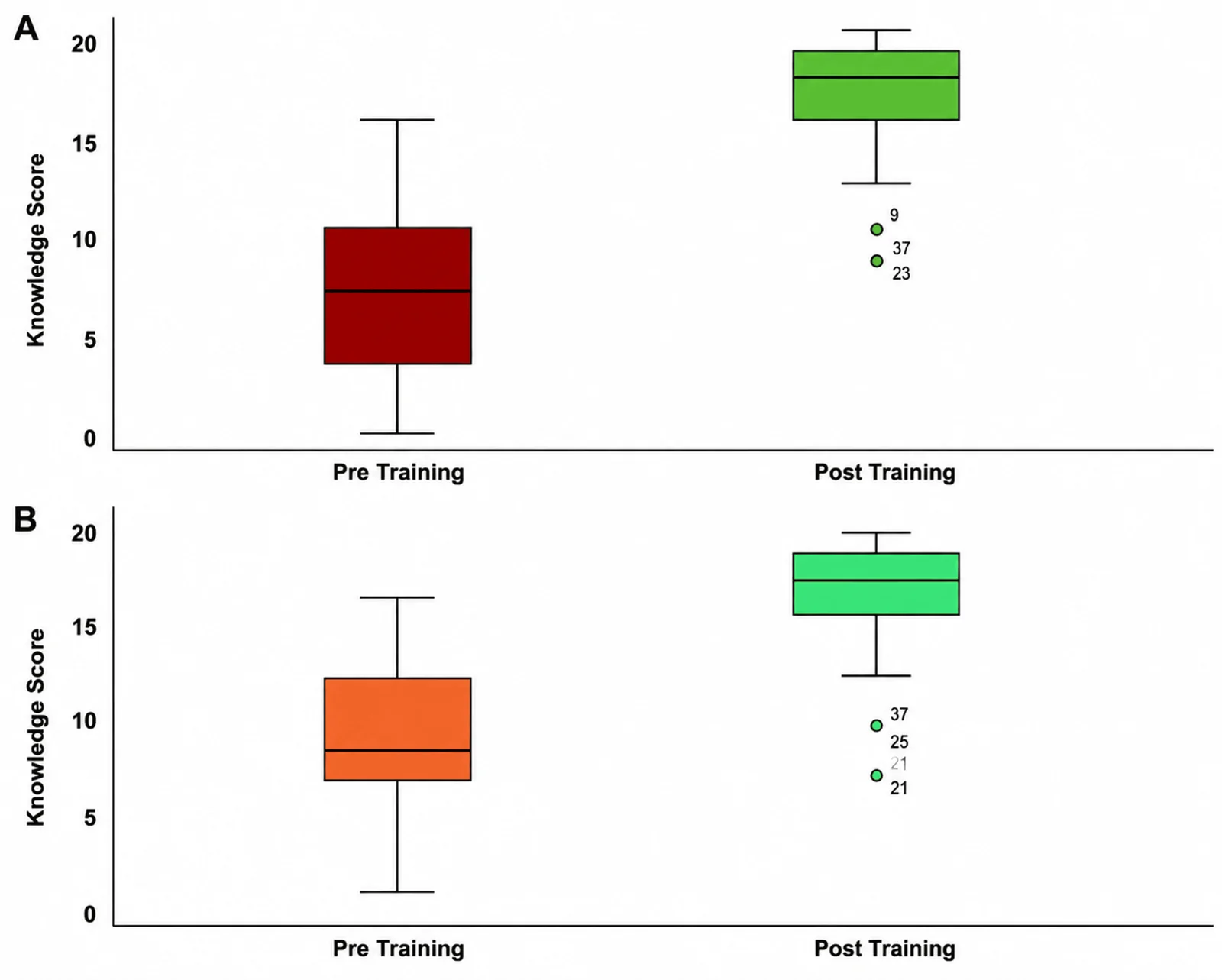
Box plots showing the distribution of pre- and post-training knowledge scores among frontline healthcare workers. Boxes represent the interquartile range (IQR), the horizontal line within each box represents the median, and the whiskers indicate the minimum and maximum values. Circles indicate outliers. A: Training 1. Japanese encephalitis among auxiliary nurse midwives (ANMs) and multi-purpose workers (MPWs) (n = 39). B: Training 2. Vector-borne disease among community health officers (CHOs) (n = 38).

The mean knowledge score increased significantly following both training sessions. Among ANMs and MPWs, the mean JE-related knowledge score increased from 7.8 ± 3.8 before training to 17.1 ± 2.8 after training (mean difference: 9.3, 95% CI: 7.7-10.9; Cohen's d = 1.9, 95% CI: 1.3-2.4; p < 0.001). Similarly, among CHOs, the mean VBD-related knowledge score increased from 8.5 ± 4.0 to 15.9 ± 2.6 (mean difference: 7.0, 95% CI: 5.6-8.3; Cohen's d = 1.7, 95% CI: 1.2-2.2; p < 0.001). The distribution of pre- and post-training JE- and VBD-related knowledge scores is shown in Figure 1.

Educational level was significantly positively correlated with improvement in JE-related knowledge (ρ = 0.400, p = 0.012). No significant correlations were observed for age, gender, years of service, place of posting, or occupation. No participant characteristic was significantly associated with change in VBD-related knowledge score among CHOs (Table 3).

**Table 3:** Spearman's rank correlation between participant characteristics and change in knowledge score following the educational intervention. Values are Spearman's rank correlation coefficients (ρ) between participant characteristics and change in knowledge score (post-training score − pre-training score). ANMs & MPWs participated in the Japanese encephalitis training, whereas CHOs participated in the vector-borne disease training. Bold indicates statistical significance (p < 0.05). Abbreviations: ANM, auxiliary nurse midwife; MPW, multi-purpose worker; CHO, community health officer; HSC, health sub centre; PHC, primary health centre; CHC, community health centre; NA, not applicable.

Participant characteristic	ANMs & MPWs (n = 39)	p-value	CHOs (n = 38)	p-value
Age (increasing)	0.036	0.828	0.217	0.190
Gender (Male ► Female)	0.138	0.402	0.130	0.436
Educational level (increasing)	0.400	0.012	0.197	0.235
Years of service (increasing)	-0.073	0.659	0.066	0.695
Place of posting (HSC ► PHC/CHC)	0.044	0.792	NA	NA
Occupation (ANM ► MPW)	-0.167	0.311	NA	NA

Multiple linear regression analysis among ANMs and MPWs identified educational level as the only independent predictor of JE-related knowledge gain (B = 1.030, 95% CI: 0.339-1.720; p = 0.005), after adjusting for age, years of service, and occupation. Gender and place of posting were excluded from the final model because of multicollinearity (Table 4).

**Table 4:** Multiple linear regression analysis of factors associated with change in knowledge score following the educational intervention among ANMs and MPWs (n = 39). Dependent variable: Change in knowledge score (post-training score − pre-training score). Adjusted R² = 0.164. Gender and place of posting were excluded from the final model because of multicollinearity (VIF = 10.617 and 12.165, respectively). Among variables retained in the model, VIF values ranged from 1.066 to 3.611 and Cook's distance from 0.000 to 0.365. Bold indicates statistical significance (p < 0.05). Abbreviations: B, unstandardized regression coefficient; SE, standard error; CI, confidence interval; ANM, auxiliary nurse midwife; MPW, multi-purpose worker; VIF, variance inflation factor.

Variable	Unstandardized coefficients		p-value	95% CI for B	
	B	SE		Lower bound	Upper bound
Age (increasing)	0.230	0.166	0.174	-0.107	0.567
Educational level (increasing)	1.030	0.340	0.005	0.339	1.720
Years of service (increasing)	-0.169	0.171	0.328	-0.516	0.178
Occupation (MPW)	-1.977	1.556	0.213	-5.139	1.185

## Discussion

This quasi-experimental study demonstrated that a one-day interactive training programme significantly improved knowledge of JE and other major VBDs among FHWs in eastern India. Mean knowledge scores more than doubled following the intervention in both training groups, with significant improvements observed across almost all assessed knowledge items. Educational level was the only independent predictor of greater knowledge gain among FHWs. A key strength of this study is the evaluation of standardized NCVBDC-aligned training programmes among different cadres of FHWs using validated assessment tools and robust statistical analyses, providing evidence to strengthen routine NCVBDC capacity-building initiatives.

Our findings are broadly aligned with findings from previous studies in India demonstrating the effectiveness of educational interventions for improving knowledge of VBDs among FHWs. A quasi-experimental study among urban link workers in Ahmedabad by Prajapati et al. [17] reported significant improvements in overall knowledge of mosquito-borne diseases and vector control measures following a single educational training session, although gaps in knowledge regarding filariasis persisted even after training. Similarly, Sahoo et al. [18] from Odisha found that accredited social health activists (ASHAs) who had received recent VBD training achieved significantly higher JE knowledge scores than those without recent training, highlighting the importance of regular refresher training for community-level health workers. Although direct comparisons should be interpreted cautiously because of differences in study settings and populations, the observed direction of effect was similar across studies. Together, these findings reinforce the value of structured educational interventions in strengthening the knowledge and preparedness of FHWs for effective VBD prevention and control.

International evidence also supports the benefits of continuous capacity-building for FHWs involved in VBD control. Togan et al. [19] from Togo reported that only a small proportion of healthcare professionals had received recent dengue training, and those who had undergone training were substantially more likely to demonstrate appropriate dengue diagnosis and management practices. Likewise, Saba et al. [20] from Pakistan found that better knowledge among mosquito control workers was associated with more appropriate vector control practices and emphasized the need for regular training to strengthen programme implementation. Hosseini et al. [21] from Iran also demonstrated significant improvements in knowledge and preventive behaviours related to leishmaniasis following structured educational interventions among volunteer health workers. Collectively, these studies suggest that well-designed educational interventions can effectively enhance competencies across diverse VBDs and healthcare settings.

Educational level was the only independent predictor of greater knowledge gain among FHWs in the present study. This finding is consistent with previous studies showing that higher educational attainment is associated with better knowledge of VBDs among healthcare workers. Togan et al. [19] reported significantly better dengue-related knowledge among university-educated healthcare professionals, while Sahoo et al. [18] observed higher JE knowledge among ASHAs with higher educational attainment and recent VBD training. Individuals with higher educational attainment may assimilate and retain new information more effectively, resulting in greater benefit from structured training programmes.

The findings have important implications for the NCVBDC. The substantial improvements observed following a brief, structured, guideline-based training programme support the continued implementation of regular refresher training for FHWs. Periodic competency assessments may help identify persistent knowledge gaps, inform targeted educational interventions, and strengthen VBD surveillance, early case detection, case management, and prevention activities at the primary healthcare level.

This study has several limitations. First, the quasi-experimental single-group pre-test-post-test design without a control group limits causal inference. Second, knowledge was assessed immediately after the training; therefore, the study evaluated short-term knowledge acquisition rather than long-term knowledge retention, and the durability of the observed improvements could not be determined. Third, although the questionnaires were developed based on the latest NCVBDC operational guidelines and demonstrated acceptable internal consistency, they were investigator-developed and were not externally validated, which may limit their broader applicability. Fourth, the study was conducted in a single district with a modest sample size of FHWs, which may limit the generalizability of the findings. Finally, although knowledge improved significantly, the study did not assess whether these gains translated into improved field practices or programme outcomes.

## Conclusions

This study demonstrated that a structured one-day interactive training programme significantly improved knowledge of JE and other major VBDs among FHWs. The broad improvements observed across almost all assessed knowledge domains and the large effect sizes suggest that standardized, guideline-based training is an effective approach for strengthening the knowledge of FHWs involved in VBD prevention and control.
